# Supplementary material for: Lymphopenia as a Biological Predictor of Outcomes in COVID-19 Patients: A Nationwide Cohort Study
Source: Cancers (Basel). 2021 Jan 26;13(3):471. doi: 10.3390/cancers13030471 (PMC7865511; doi:10.3390/cancers13030471)
Supplement: Supplementary file 1 [file cancers-13-00471-s001.pdf]

cancers

MDPI

Article

Lymphopenia as a Biological Predictor of Outcomes in COVID-19 Patients: A Nationwide Cohort Study

Jongmin Lee [1,2,†], Sung-Soo Park [2,3,†], Tong Yoon Kim [2,3,†], Dong-Gun Lee [2,4,*] and Dong-Wook Kim [2,3,*]

1 Division of Pulmonary, Allergy and Critical Care Medicine, Department of Internal Medicine, The Catholic University of Korea, Seoul 06591, Korea; dibs01@catholic.ac.kr
2 Catholic Hematology Hospital, College of Medicine, The Catholic University of Korea, Seoul 06591, Korea; sspark@catholic.ac.kr (S.-S.P.); TYK@catholic.ac.kr (T.Y.K.)
3 Division of Hematology, Department of Internal Medicine, College of Medicine, The Catholic University of Korea, Seoul 06591, Korea
4 Division of Infectious Diseases, Department of Internal Medicine, College of Medicine, The Catholic University of Korea, Seoul 06591, Korea
* Correspondence: symonlee@catholic.ac.kr (D.-G.L.); dwkim@catholic.ac.kr (D.-W.K.); Tel.: +82-2-2258-6003 (D.-G.L.); +82-2-2258-7030 (D.-W.K.); Fax: +82-2-535-2494 (D.-G.L.); +82-2-593-2522 (D.-W.K.)
† The first author: J.L., S.-S.P. and T.Y.K. contributed equally to this work as first authors.

Supplementary

Citation: Lee, J.; Park, S.-S.; Kim, T.Y.; Lee, D.-G.; Kim, D.-W. Lymphopenia as a Biological Predictor of Outcomes in COVID-19 Patients: A Nationwide Cohort Study. *Cancers* **2021**, *13*, 471. https://doi.org/10.3390/cancers13030471

Academic Editors: Gabriella D'Orazi and Mara Cirone
Received: 28 November 2020
Accepted: 20 January 2021
Published: 26 January 2021

Publisher's Note: MDPI stays neutral with regard to jurisdictional claims in published maps and institutional affiliations.

Copyright: © 2021 by the authors. Licensee MDPI, Basel, Switzerland. This article is an open access article distributed under the terms and conditions of the Creative Commons Attribution (CC BY) license (http://creativecommons.org/licenses/by/4.0/).

Table S1. Univariable analysis of factors associated with mortality.

Variable	Survival outcome (total N = 770)		*p-value*
	Death (n = 162)	Alive (n = 608)	
Age			<0.001
< 40 years, no (%)	1 (1.7%)	59 (98.3%)	
40 - 59 years, no (%)	13 (6.7%)	182 (93.3%)	
≥ 60 years, no (%)	148 (28.7%)	367 (71.3%)	
**Gender**, no (%)			0.999
Female, no (%)	67 (21.1%)	250 (78.9%)	
Male, no (%)	95 (21.0%)	358 (79.0%)	
**Systolic blood pressure, baseline,** missing n=2 / n=3			0.016
< 140 mmHg, no (%)	84 (17.8%)	388 (82.2%)	
≥ 140 mmHg, no (%)	76 (25.9%)	217 (74.1%)	
**Diastolic blood pressure, baseline,** missing n=2 / n=3			0.331
< 80 mmHg, no (%)	80 (19.5%)	331 (80.5%)	
≥ 80 mmHg, no (%)	80 (22.6%)	274 (77.4%)	
**Heart rate, baseline**			0.002
< 110 / min, no (%)	131 (19.3%)	547 (80.7%)	
≥ 110 / min, no (%)	31 (33.7%)	61 (66.3%)	
**Body temperature, baseline**			0.943
< 38.0 ℃, no (%)	133 (19.3%)	503 (80.7%)	
≥ 38.0 ℃, no (%)	29 (33.7%)	105 (66.3%)	
**Baseline presentation**			
Sputum (+), no (%)	54 (21.4%)	198 (78.6%)	0.928
Fatigue (+), no (%)	13 (31.7%)	28 (68.3%)	0.127
Dyspnea (+), no (%)	86 (37.4%)	144 (62.6%)	<0.001
Mental disturbance (+), no (%)	16 (80%)	4 (20%)	<0.001
Nausea/vomiting (+), no (%)	11 (18.6%)	48 (81.4%)	0.762
Diarrhea (+), no (%)	15 (13.2%)	99 (86.8%)	0.035
**Comorbidity**			
Treating cancer (+), no (%)	19 (34.5%)	36 (65.5%)	0.017
Diabetes (+), no (%)	68 (32.5%)	141 (67.5%)	<0.001
Hypertension (+), no (%)	102 (30.3%)	235 (69.7%)	<0.001
Chronic cardiac disease (+), no (%)	26 (33.3%)	52 (66.7%)	0.008
Chronic pulmonary disease (+), no (%)	11 (37.9%)	18 (62.1%)	0.041
Chronic renal disease (+), no (%)	14 (48.3%)	15 (51.7%)	0.001
Chronic hepatic disease (+), no (%)	5 (14.7%)	29 (85.3%)	0.477
Autoimmune disease (+), no (%)	3 (23.1%)	10 (76.9%)	0.999
Dementia (+), no (%)	64 (48.1%)	69 (51.9%)	<0.001
**Baseline hemogram**			
Hemoglobin			<0.001
≥ 12.5 g/dL, no (%)	54 (13.1%)	358 (86.9%)	
< 12.5 g/dL, no (%)	108 (30.2%)	250 (69.8%)	
Absolute lymphocyte counts			<0.001
Group III, ≥ 1,000 /mm^3, no (%)	43 (13.0%)	287 (87.0%)	
Group II, ≥ 500 - < 1,000 /mm^3, no (%)	75 (22.7%)	255 (77.3%)	
Group I, < 500 /mm^3, no (%)	44 (40.0%)	66 (60.0%)	
Platelet, missing n=2 / n=83, no (%)			0.003
≥ 100,000 /mm^3, no (%)	141 (19.7%)	573 (80.3%)	
< 100,000 /mm^3, no (%)	21 (37.5%)	35 (62.5%)	

Table S2. Comparisons of studies having data regarding mortality and proportion of lymphopenia defined as absolute lymphocyte count < 1,000 /mm^3.

Study	Number	Proportion of lymphopenia of total cohort (%)	Mortality	Remark
Yang X et al.	52	84.6%	61.5%	China data (Wuhan)
Richardson S et al.	5,645	60.0%	21.0%	America data (New York)

Current study	770	57.1%	21.0%	Korean data (Nationwide cohort)
Acharya D et al.	324	19.1%	7.4%	Korean data (Gyeongsangbuk-do)
Moon S-S et al.	352	17.9%	6.8%	Korean data (Gyeongsangbuk-do)
